# Assessment of Melatonergics in Prevention of Delirium: A Systematic Review and Meta-Analysis

**DOI:** 10.3389/fneur.2020.00198

**Published:** 2020-04-07

**Authors:** Yibing Zhu, Zhiming Jiang, Huibin Huang, Wen Li, Chao Ren, Renqi Yao, Yang Wang, Yongming Yao, Wei Li, Bin Du, Xiuming Xi

**Affiliations:** ^1^Medical Research and Biometrics Center, National Center for Cardiovascular Diseases, Fuwai Hospital, Chinese Academy of Medical Sciences and Peking Union Medical College, Beijing, China; ^2^Department of Critical Care Medicine, Fuxing Hospital, Capital Medical University, Beijing, China; ^3^Department of Respiratory Medicine, Qilu Hospital of Shandong University, Jinan, China; ^4^School of Medicine, Shandong University, Jinan, China; ^5^Department of Pulmonary and Critical Care Medicine, The First Affiliated Hospital of Shandong First Medical University, Jinan, China; ^6^Medical ICU, Peking Union Medical College Hospital, Peking Union Medical College and Chinese Academy of Medical Sciences, Beijing, China; ^7^Department of Critical Care Medicine, Beijing Tsinghua Chang Gung Hospital, Beijing, China; ^8^School of Medicine, Nankai University, Tianjin, China; ^9^Trauma Research Center, Fourth Medical Center of the Chinese PLA General Hospital, Beijing, China; ^10^State Key Laboratory of Kidney Disease, The Chinese PLA General Hospital, Beijing, China; ^11^Department of Burn Surgery, Changhai Hospital, The Second Military Medical University, Shanghai, China

**Keywords:** melatonin, delirium, prevention, critical care medicine, systematic review

## Abstract

**Background:** Delirium is a commonly found comorbidity in hospitalized patients and is associated with adverse outcomes. Melatonin is an endogenous hormone that exerts multiple biological effects, mainly in regulating diurnal rhythms and in inflammatory process and immune responses. We aimed to assess the efficacy of exogenous melatonergics in the prevention of delirium.

**Methods:** We conducted a search to identify relevant randomized controlled studies (RCTs) in PubMed, Cochrane Library, and EMBASE databases that had been published up to December 2019. Hospitalized adult patients administered melatonergics were included. The primary outcome measure was the incidence of delirium. The secondary outcome measure was the length of stay in intensive care unit (ICU-LOS). The pooled effects were analyzed as the risk ratio (RR) for delirium incidence, weighted mean difference (WMD) for ICU-LOS, and 95% confidence intervals (CIs).

**Results:** Nine RCTs with 1,210 patients were included. The forest plots showed that melatonergics were associated with a decreasing incidence of delirium (RR, 0.51; 95% CI, 0.30–0.85; *I*^2^ = 70%; *p* = 0.01). There was no significant difference in ICU-LOS (WMD, −0.08; 95% CI, −0.19–0.03; *I*^2^ = 0; *p* = 0.17).

**Conclusion:** Administration of exogenous melatonergics to hospitalized patients seems to be associated with a decreasing incidence of delirium.

**PROSPERO registration number:** CRD42019138863.

## Background

Delirium is a complex neuropsychiatric syndrome characterized by cognitive impairment and attentional deficits (1). Delirium in the intensive care unit (ICU) has a high incidence (30–60%) (2–4) and is strongly associated with adverse outcomes, such as increased mortality, prolonged length of stay in ICU (ICU-LOS), increased costs, and long-term cognitive sequelae (5). As increasingly recognized, delirium has become one of the most concerning problems for intensive care physicians. Although delirium appears to have a high incidence and is associated with adverse outcomes, clinical strategies have been very limited (6).

Melatonin is a neurohormone that exerts multiple biological effects, mainly in regulating diurnal rhythms and in modulating inflammatory as well as immune responses (7, 8). Abundant evidence has indicated that decreasing melatonin levels are linked with delirium (9). Melatonergics include melatonin and other melatonin agonists such as ramelteon. Whether supplementation with exogenous melatonin and melatonin agonists could reduce the risk of delirium remains uncertain. Several randomized controlled studies (RCTs) have been conducted related to this issue. Taking Sultan and colleagues' study (10) as an example, 300 elderly patients undergoing hip arthroplasty were randomly assigned to one of four groups; these groups were administered with melatonin, midazolam, clonidine, or no medication, respectively. The results of this study showed that melatonin was associated with a significant reduction in the incidence of delirium compared with the control group, as well as the other two parallel groups. However, a meta-analysis including four RCTs indicated no significant difference (11). Since then, several well-designed RCTs have been conducted (12–16). A recent meta-analysis evaluated delirium as a subset but included only three RCTs for this result (17). With the updated results (12–16), we aimed to conduct a meta-analysis in attention to reevaluate the efficacy of melatonergics in the prevention of delirium.

## Methods

### Study Registration

The protocol has been registered on the PROSPERO (registration no. CRD42019138863) based on the Preferred Reporting Items for Systematic review and Meta-analysis Protocols guidelines (18).

### Search Strategy and Selection Criteria

Three electronic databases (PubMed, Cochrane Library, and EMBASE) were searched without language restriction to identify RCTs published from 1960 to December 2019. Multiple keywords including “melatonin,” “melatonergic,” or “ramelteon” combined with “delirium” were developed for the search strategy. The reference lists were searched manually for potentially relevant articles.

Studies meeting the following criteria were included: (1) the study was designed as an RCT; (2) the study subjects consisted of hospitalized adults (aged ≥18 years); (3) melatonin or melatonin agonists were administered; and (4) outcome measures included delirium incidence.

Studies meeting the following criteria were excluded: (1) publications available only in abstract form or as meeting reports or (2) studies of suboptimal quality (modified Jadad score 0–4) (19).

### Data Extraction and Quality Assessment

Two reviewers (J.Z.M. and Z.Y.B.) independently extracted data to fill in a predesigned form including the characteristics of the studies, year of publication, demographics and baseline of subjects, intervention, and outcomes. Two reviewers (Z.Y.B. and W.Y.) independently rated the quality of the RCTs and extracted the items for the risk-of-bias assessment (19). Disagreements between reviewers were resolved by two experts (X.X.M. and Y.Y.M.).

### Outcome and Statistical Analysis

The primary outcome measure was the incidence of delirium. The pooled effects were analyzed as risk ratio (RR) using the Mantel–Haenszel technique and 95% confidence intervals (CIs). Sensitivity analyses of the primary outcome were conducted. Subgroup analyses of the primary outcome included (a) differed melatonergics, including melatonin and ramelteon; (b) different age groups, including elderly subjects (mean age, >60 years), middle-aged subjects (mean age, 40–60 years), and younger subjects (mean age, <40 years); (c) different ICU types, including surgical ICU, medical ICU, and mixed ICU; and (d) different delirium assessment methods including the Confusion Assessment Method for the ICU (CAM-ICU), the Diagnostic and Statistical Manual of Mental Disorders (*DSM-IV*), Abbreviated Mental Test (AMT), the Confusion Assessment Method (CAM), the nurse observation, and the methods not reported. The secondary outcome measure was ICU-LOS. The pooled ICU-LOS was measured in days and analyzed using the weighted mean difference (WMD) and 95% CI. If high clinical or statistical heterogeneity was observed, a random-effects model was chosen. Otherwise, a fixed-effects model was used. The *I*^2^ statistic was used to estimate statistical heterogeneity (*I*^2^ < 30% as low heterogeneity, *I*^2^ of 30–70% as medium heterogeneity, and *I*^2^ > 70% as high heterogeneity). *P* < 0.05 was considered to be statistically significant.

## Results

### Study Selection

**Figure 1:**
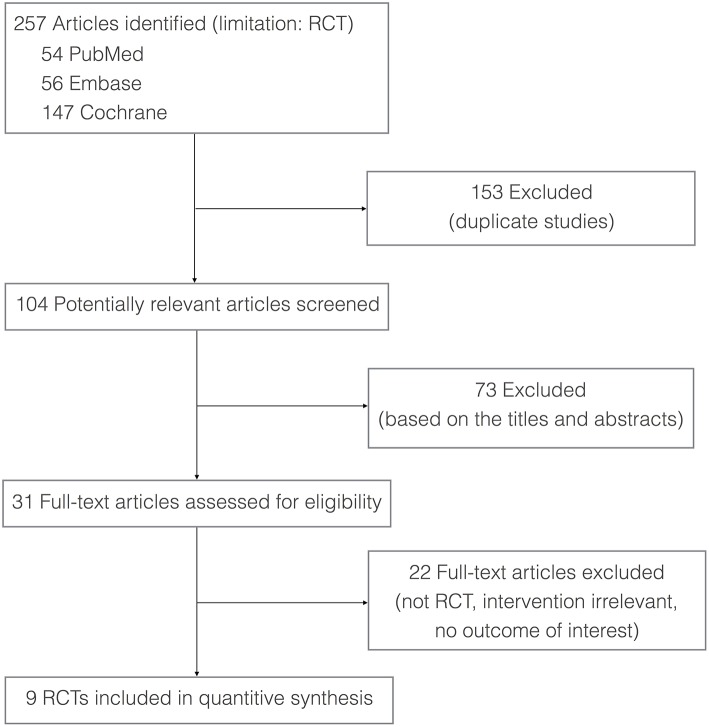
Flow diagram of the process for identification of the included studies.

A total of 257 potentially relevant articles were identified according to the search strategy. The full text of 31 articles was obtained after screening the titles/abstracts. Twenty-two studies failed to meet the inclusion criteria; therefore, nine studies were included in this meta-analysis. Figure 1 presents the process of study selection.

### Study Characteristics and Quality

**Figure 2:**
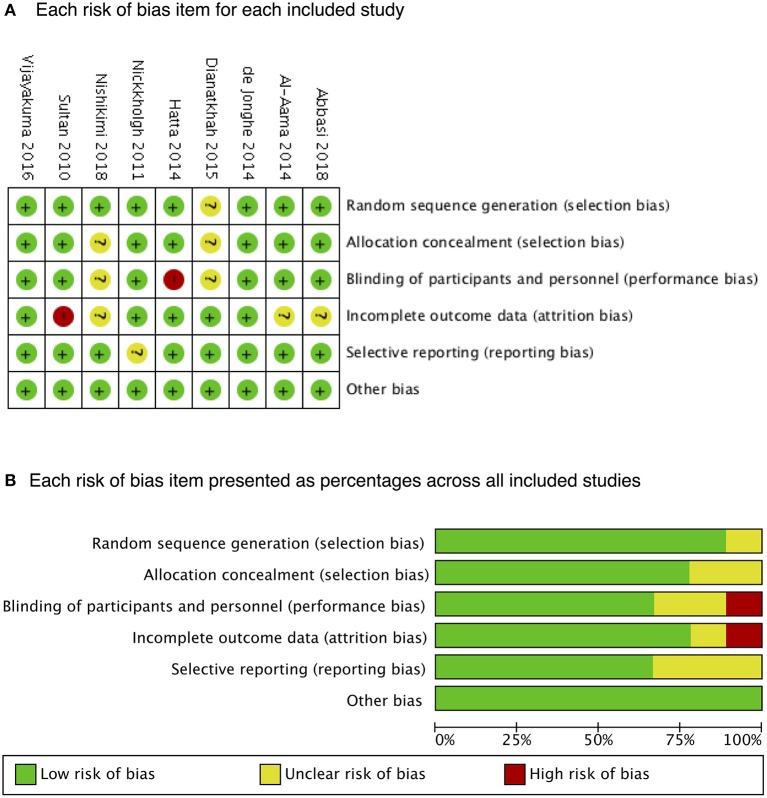
Risk of bias. A summary of **(A)** each risk of bias item for each included study and **(B)** each risk-of-bias item presented as percentages across all included studies.

Melatonin was administered in seven RCTs (10, 12–14, 16, 20, 21) with different doses. Ramelteon was administered in the other two (15, 22). The subjects of six RCTs (10, 13, 15, 20–22) were elderly (mean age, >60 years), whereas they were apparently younger (mean age, <40 years) in the other (12). Table 1 presents the characteristics of the included studies. The nine RCTs were of high quality. Figure 2 presents the risk-of-bias items for each study.

**Table 1:** Characteristics of included studies.

**References**	**Participants**	**Sample size (M/C)**	**mean age (M/C)**	**Mean APACHE II score (M/C)**	**Interventions**	**Measures**	**Delirium incidence (%)**	**Outcomes**
Sultan (10)	Elderly subjects undergoing hip arthroplasty	53/49	70.4/72.3	14.6/13.5	MT (5 mg) vs. no medication administered preoperative bedtime, 90 min preoperation, and 3 nights postoperation	Delirium assessed by AMT	11	MT group with lower delirium rate
Nickkholgh et al. (16)	Middle age subjects undergoing liver surgery	18/18	59/56	NR	MT (50 mg/kg BW) vs. placebo administered on the day of surgery	NR	3	MT group with lower delirium rate
Al-Aama et al. (20)	Elderly subjects on internal medicine service	61/61	84.3/86.4	NR	MT (0.5 mg) vs. placebo given flexibly between 6 pm and midnight	Delirium assessed by CAM; delirium severity assessed by MDAS	34	MT group with lower delirium rate; no difference in delirium severity
Hatta et al. (22)	Elderly subjects in the ICU and on internal medicine wards	33/34	78.2/78.3	13.5/14.6	Ramelteon (8 mg) vs. placebo at 9 pm for up to seven nights	Delirium assessed by *DSM-IV*; delirium severity assessed by DSR-98	68	Ramelteon group with lower odds of delirium; ramelteon group with longer time to delirium
de Jonghe et al. (21)	Elderly subjects undergoing hip surgery	186/192	84.1/83.4	NR	MT (3 mg) vs. placebo in the evening for 5 days	Delirium assessed by *DSM-IV*	28	MT group with higher delirium rate
Dianatkhah et al. (13)	Elderly subjects undergoing CABG surgery	66/71	60.0/62	NR	MT (3 mg) vs. oxazepam 1 h before sleep time from 3 days before surgery to discharge	Delirium assessed by nurse records	10	MT group with lower delirium rate
Vijayakumar et al. (12)	Younger subjects with lesser comorbidity	26/30	36.9/38	10.2/8.6	MT (3 mg) vs. placebo at 9 pm throughout the ICU stay	Delirium assessed by CAM-ICU	28	MT group with a longer delirium free day; MT group with lower incidence
Abbasi et al. (14)	Middle age subjects in mixed ICU	67/70	52.5/49.9	8.1/4.3	MT (3 mg) vs. placebo at 9 pm for 5 days	Delirium assessed by CAM-ICU	3	MT group with higher delirium rate
Nishikimi et al. (15)	Elderly subjects on internal medicine service	45/43	68/68	23.9/23.9	Ramelteon (8 mg) vs. placebo at 8 pm every day until discharge from ICU	Delirium assessed by CAM-ICU	35	Ramelteon group with lower incidence of delirium

*AMT, Abbreviated Mental Test; APACHE II, Acute Physiology and Chronic Health Evaluation II; CABG, coronary artery bypass graft; CAM, Confusion Assessment Method; CAM-ICU Confusion Assessment Method for the ICU; DSM-IV, Diagnostic and Statistical Manual of Mental Disorders, Fourth Edition; DSR-98, Delirium Rating Scale–revised-98; ICU, intensive care unit; M/C, melatonin or melatonin agonist group/control group; MDAS, Memorial Delirium Assessment Scale; MT, melatonin; NR, not reported*.

### Outcomes

**Figure 3:**
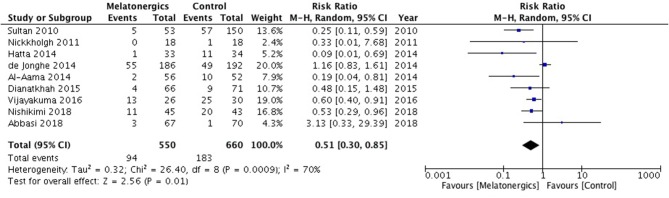
Forest plot. Administration of melatonergics was associated with a reduction of the incidence of delirium.

Nine RCTs with 1,210 patients were involved to analyze the incidence of delirium. Forest plots showed that melatonergics were associated with a decreasing incidence of delirium (RR, 0.51; 95% CI, 0.30–0.85; *I*^2^ = 70%; *p* = 0.01; Figure 3). The statistical heterogeneity of the data was high. In the sensitivity analyses, the difference between the melatonergics and the control groups remained significant when we excluded any single RCT (Table 2). In the subgroup analyses, melatonergics showed an association with lower delirium incidence in the subgroup of elderly patients, younger patients, medical ICU patients, delirium screening methods of CAM-ICU, AMT, and CAM. In the melatonin, ramelteon, middle-aged, surgical and mixed ICU, delirium screening methods of *DSM-IV*, nurse observation, and methods not reported subgroups, there was no significant difference (Table 3).

**Table 2:** Sensitivity analyses on delirium incidence.

**References**	**Patients remaining (M/C)**	**Events remaining (M/C)**	**RR (95% CI)**	***I*^2^ (%)**	***p***
Sultan (10)	496/510	89/126	0.58 (0.35–0.97)	64	0.04
Nickkholgh et al. (16)	532/642	94/182	0.51 (0.30–0.87)	73	0.01
Hatta et al. (22)	517/626	93/172	0.56 (0.34–0.93)	68	0.003
de Jonghe et al. (21)	364/468	39/134	0.43 (0.27–0.69)	41	0.0005
Al-Aama et al. (20)	494/608	92/173	0.56 (0.33–0.94)	69	0.03
Dianatkhah et al. (13)	484/589	90/174	0.51 (0.29–0.89)	73	0.02
Vijayakumar et al. (12)	524/630	81/158	0.47 (0.23–0.93)	73	0.03
Nishikimi et al. (15)	487/599	83/162	0.50 (0.26–0.94)	76	0.03
Abbasi et al. (14)	483/590	91/182	0.47 (0.28–0.79)	72	0.005

*CI, confidence interval; C, control group; M, melatonergics group; No., number; RR, risk ratio*.

**Table 3:** Subgroup analyses on delirium incidence.

	**Group**	**Reference**	**Patient no. (M/C)**	**Event (M/C)**	**RR (95%CI)**	***I*^2^ (%)**	***p***
Melatonergics	Melatonin	10, 12, 13, 14, 18, 20, 21	472/583	82/152	0.56 (0.31–1.02)	71	0.06
	Ramelteon	15, 22	78/77	12/31	0.28 (0.05–1.61)	67	0.15
Age	Elderly	10, 13, 15, 20, 21, 22	493/542	78/156	0.41 (0.19–0.86)	80	0.02
	Middle	14, 18	85/88	3/2	1.35 (0.16–11.35)	23	0.78
	Younger	12	26/30	13/25	0.60 (0.40–0.91)	NA	0.02
ICU type	Medical	15, 20, 22	134/129	14/41	0.27 (0.09–0.82)	55	0.02
	Surgical	10, 13, 18, 21	323/431	64/116	0.53 (0.19–1.46)	78	0.22
	Mixed	12, 14	93/100	16/26	0.98 (0.20–4.65)	56	0.98
Assessment tools	CAM-ICU	12, 14, 15	138/143	27/46	0.60 (0.41–0.90)	14	0.01
	*DSM-IV*	21, 22	219/226	56/60	0.40 (0.03–5.02)	85	0.47
	AMT	10	53/150	5/57	0.25 (0.11–0.59)	NA	0.001
	CAM	20	56/52	2/10	0.19 (0.04–0.81)	NA	0.02
	Nurse	13	66/71	4/9	0.48 (0.15–1.48)	NA	0.20
	NR	18	18/18	0/1	0.33 (0.01–7.68)	NA	0.49

*C, control group; CAM-ICU, Confusion Assessment Method for the ICU; *DSM-IV, Diagnostic and Statistical Manual of Mental Disorders, Fourth Edition*; ICU, intensive care unit; M, melatonergics group; NA, not applicable; No., number; RR, risk ratio*.

**Figure 4:**
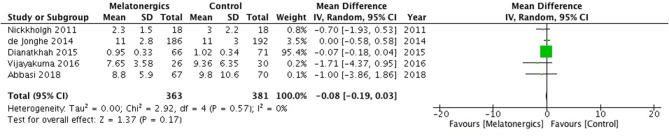
Forest plot. There was no significant difference in the length of stay in ICU.

For the secondary outcome, the ICU-LOS in the melatonergics group was numerically shorter than that in the control group, with no significant difference (WMD, −0.08; 95% CI, −0.19–0.03; *I*^2^ = 0%; *p* = 0.17; Figure 4).

## Discussion

The results of the present meta-analysis indicated that melatonergics were associated with a decreasing incidence of delirium in critically ill patients. Based on the encouraging results of the primary RCTs (10, 12, 20, 22), suggesting that administration of melatonin and melatonin agonists might be a promising medication for the prevention of delirium in at-risk populations, our meta-analysis provided advanced evidence. These encouraging results were considered to be quite reasonable. First, sleep deprivation is a crucial risk factor for delirium (21). Exogenous melatonergics could be associated with improvement in sleep quality and prolongation of sleep duration in some settings. Meanwhile, a reduction in melatonin levels is associated with the development of delirium. Thus, supplementation with exogenous melatonin can remedy disordered melatonin levels (23). Second, delirium may be precipitated in some patients by inflammation of the central nervous system. Melatonin is an anti-inflammatory drug, and antioxidants function protectively during the inflammatory response (24). Third, delirium occurs commonly as a side effect of benzodiazepines, the most commonly used sedatives in the ICU (25). Administration of melatonergics to hypnosis could reduce the dosage of benzodiazepines accordingly.

Although the ICU-LOS in the melatonergics group was numerically shorter than that in the control group, there was no significant difference. The reasons could be as follows. First, there were only five RCTs with 744 patients included in this outcome measure. The result should be interpreted more cautiously because of the limited sample size. Second, because the context of the ICU is linked to the delirium onset and is bad for delirium recovery, physicians might tend to discharge patients with delirium or a high risk of delirium to general wards more aggressively. This confounder makes the association between delirium and ICU-LOS more obscure and hard to interpret.

### Strengths and Limitations

There are strengths to our study. First, in comparison to another meta-analysis (11), we excluded previously diagnosed delirium [according to what the authors reported (21)] and analyzed incident delirium to reduce confounding factors and clinical heterogeneity. Second, our sensitivity analyses showed steady and consistent results, which increased the grade of evidence.

There are also limitations to our study. First, clinical heterogeneity was obvious between the RCTs due to varied age, Acute Physiology and Chronic Health Evaluation II score and comorbidity of subjects, varied dosage of melatonergics in intervention strategy, and different methods for delirium assessment. The assessment of delirium has long been challenging for ICU physicians. Without gold standard for diagnosis, different delirium scales have been used (6). The heterogeneity in assessment methods added difficulty in the interpretation of the findings. The 2013 clinical practice guidelines for the management of pain, agitation, and delirium in adult patients in the ICU recommended CAM-ICU, the most valid monitoring tool so far (6). After that, the latest RCTs (12, 14, 15) used CAM-ICU as the assessment tool. More experience using CAM-ICU to screen delirium is needed for future studies and clinical practice. Second, although nine RCTs were involved, the overall sample size was not large. In addition, the small number of RCTs (<10) also added difficulty in estimating publication bias. Potential negative unpublished studies may be missing, resulting in overoptimism in our conclusion. Additionally, the effect of melatonergics in the treatment of delirium was not studied in the present meta-analysis, which is of great value and needs to be further studied.

## Conclusions

Exogenous melatonergics seem to be associated with a decreased incidence of delirium. No significant difference in ICU-LOS was identified. Additional studies are needed to further evaluate the efficacy and safety of melatonin in preventing delirium.

## Author Contributions

ZJ, YZ, and HH searched the scientific literature and drafted the manuscript, collect the data, and performed statistical analyses. RY, CR, YW, YY, and WenL polished and revised the manuscript. WeiL, XX, and BD contributed to conception, design, data interpretation, manuscript revision for critical intellectual content, and supervision of the study. All authors read and approved the manuscript.

### Conflict of Interest

The authors declare that the research was conducted in the absence of any commercial or financial relationships that could be construed as a potential conflict of interest.

## Abbreviations

AMT: Abbreviated Mental Test
CAM: Confusion Assessment Method
CAM-ICU: Confusion Assessment Method for the Intensive Care Unit
CIs: confidence intervals
ICU: intensive care unit
ICU-LOS: length of stay in intensive care units
OR: odds ratio
RCTs: randomized controlled studies
RR: risk ratio
WMD: weighted mean difference.
